# Supplementary material for: Identification of photoperiod-induced specific miRNAs in the adrenal glands of Sunite sheep (Ovis aries)
Source: Front Vet Sci. 2022 Jul 22;9:888207. doi: 10.3389/fvets.2022.888207 (PMC9354845; doi:10.3389/fvets.2022.888207)
Supplement: Supplementary file 1 [file Data_Sheet_1.ZIP › Supplementary file/miRDeep2 file.zip!/miRDeep2 file/Novel_Structure/SP42A1/Novel_664.pdf]

Provisional ID : NC_019481.2_25088
Score total : 368
Score for star read(s) : 3.9
Score for read counts : 360.6
Score for mfe : 2
Score for randfold : 1.6
Score for cons. seed :
Total read count : 719
Mature read count : 692
Loop read count : 0
Star read count : 27

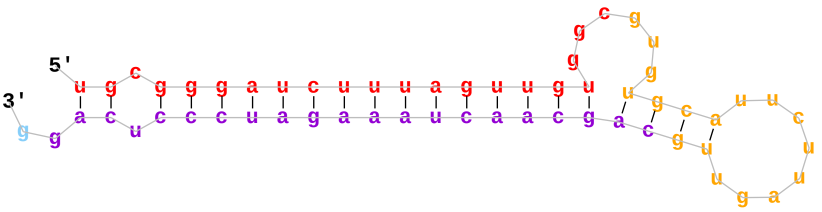


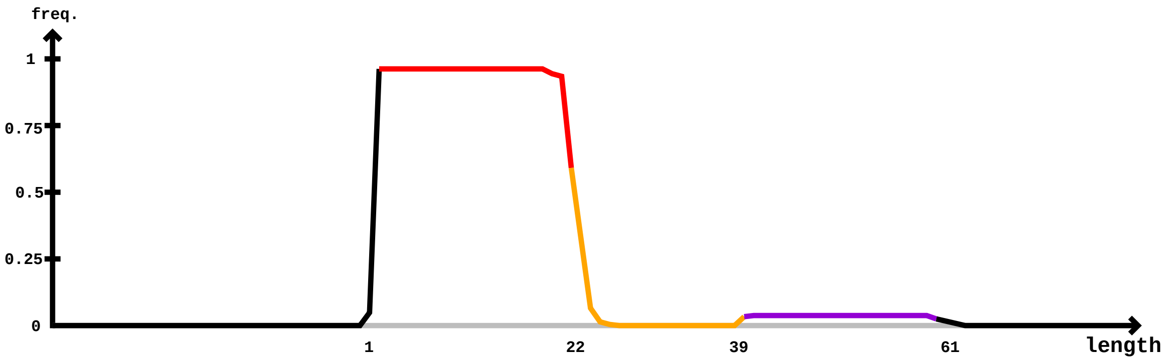


Mature Star

```
5'- aauaacccauaaaagcagccgacguugcggcaugcgggaucuuuaguuguggcgugugcauucuuaguugcagcaacuaaagaucccucaggcugcaaugaagaucagaga -3'  obs
    aauaacccauaaaagcagccgacguugcggcaugcgggaucuuuaguuguggcgugugcauucuuaguugcagcaacuaaagaucccucaggcugcaaugaagaucagaga       exp
    ...................(..(((((((((.((.(((((((((((((((......((((........))))))))))))))))))).)).)))))))))..)........       reads  mm  sample
    ...............................augcgggaucuuuaguugu.............................................................       1      0   seq
    ...............................augAgggaucuuuaguugu.............................................................       1      1   seq
    ...............................augcgggaucuuuaguugugU...........................................................       2      1   seq
    ...............................augcgggaucuuuaguuguggU..........................................................       27     1   seq
    ...............................augcgggaucuuuaguuguggcC.........................................................       1      1   seq
    ...............................augcAggaucuuuaguuguggcgu........................................................       1      1   seq
    ...............................augcgggaucuuuaguuguggcAu........................................................       2      1   seq
    ................................ugcgggaucuuuaguugA.............................................................       5      1   seq
    ................................ugcgggaucuuuaguugC.............................................................       6      1   seq
    ................................ugcgggaucuuuaguuguA............................................................       7      1   seq
    ................................ugcgggaucuuuaguugugg...........................................................       223    0   seq
    ................................ugcAggaucuuuaguugugg...........................................................       5      1   seq
    ................................uAcgggaucuuuaguugugg...........................................................       1      1   seq
    ................................ugcgggaucuuuaguugugU...........................................................       15     1   seq
    ................................ugcgggaucuuuaguugCgg...........................................................       1      1   seq
    ................................ugGgggaucuuuaguuguggc..........................................................       2      1   seq
    ................................ugcgAgaucuuuaguuguggc..........................................................       1      1   seq
    ................................ugcUggaucuuuaguuguggc..........................................................       3      1   seq
    ................................ugcAggaucuuuaguuguggc..........................................................       5      1   seq
    ................................ugcgggaucuuuaguuguggU..........................................................       280    1   seq
    ................................ugcgggaucuuuaguugCggc..........................................................       3      1   seq
    ................................uAcgggaucuuuaguuguggc..........................................................       2      1   seq
    ................................ugUgggaucuuuaguuguggc..........................................................       3      1   seq
    ................................ugcgggaucuuuaguugugAc..........................................................       1      1   seq
    ................................ugcUggaucuuuaguuguggcg.........................................................       5      1   seq
    ................................uAcgggaucuuuaguuguggcg.........................................................       7      1   seq
    ................................ugcgggaucuuuaguuguggUg.........................................................       20     1   seq
    ................................ugcgggaucuuuagCuguggcg.........................................................       8      1   seq
    ................................ugcgggGucuuuaguuguggcg.........................................................       5      1   seq
    ................................ugcgAgaucuuuaguuguggcg.........................................................       5      1   seq
    ................................ugcgggaucuuuagCuguggcgu........................................................       2      1   seq
    ................................ugcgggGucuuuaguuguggcgu........................................................       6      1   seq
    ................................uAcgggaucuuuaguuguggcgu........................................................       6      1   seq
```

```
                                Mature                              Star
aauaacccauaaaagcagccgacguugcggcaugcgggaucuuuaguuguggcgugugcauucuuaguugcagcaacuaaagaucccucaggcugcaaugaagaucagaga
................................ugcgggaucuuuaguuguggUgu........................................................    9    1    seq
................................ugcgAgaucuuuaguuguggcgu........................................................    9    1    seq
................................ugcUggaucuuuaguuguggcgu........................................................    2    1    seq
................................ugcgggaucuuuaguuguggcgug.......................................................    7    0    seq
................................ugcgggaucuuuaguuguggcgugu......................................................    3    0    seq
......................................................................cagcaacuaaagaucccuca.....................    9    0    seq
......................................................................cagcaacuaaagaucccucag....................   15    0    seq
.......................................................................agcaacuaaagaucccucaggc..................    3    0    seq
```
